# Prenatal Phthalate Exposures and Childhood Fat Mass in a New York City Cohort

**DOI:** 10.1289/ehp.1509788

**Published:** 2015-08-25

**Authors:** Jessie P. Buckley, Stephanie M. Engel, Michelle A. Mendez, David B. Richardson, Julie L. Daniels, Antonia M. Calafat, Mary S. Wolff, Amy H. Herring

**Affiliations:** 1 Department of Epidemiology, and; 2 Department of Nutrition, University of North Carolina at Chapel Hill, Chapel Hill, North Carolina, USA; 3 Division of Laboratory Sciences, National Center for Environmental Health, Centers for Disease Control and Prevention, Atlanta, Georgia, USA; 4 Department of Community and Preventative Medicine, Mount Sinai School of Medicine, New York, New York, USA; 5 Department of Biostatistics and Carolina Population Center, University of North Carolina at Chapel Hill, Chapel Hill, North Carolina, USA

## Abstract

**Background:**

Experimental animal studies and limited epidemiologic evidence suggest that prenatal exposure to phthalates may be obesogenic, with potential sex-specific effects of phthalates having anti-androgenic activity.

**Objectives:**

We aimed to assess associations between prenatal phthalate exposures and childhood fat mass in a prospective cohort study.

**Methods:**

We measured phthalate metabolite concentrations in third-trimester maternal urine in a cohort of women enrolled in New York City between 1998 and 2002 (n = 404). Among 180 children (82 girls and 98 boys), we evaluated body composition using a Tanita scale at multiple follow-up visits between ages 4 and 9 years (363 total visits). We estimated associations of standard deviation differences or tertiles of natural log phthalate metabolite concentrations with percent fat mass using linear mixed-effects regression models with random intercepts for repeated outcome measurements. We assessed associations in multiple metabolite models and adjusted for covariates including prepregnancy body mass index, gestational weight gain, maternal smoking during pregnancy, and breastfeeding.

**Results:**

We did not observe associations between maternal urinary phthalate concentrations and percent body fat in models examining continuous exposures. Fat mass was 3.06% (95% CI: –5.99, –0.09%) lower among children in the highest tertile of maternal urinary concentrations of summed di(2-ethylhexyl) phthalate (ΣDEHP) metabolites than in children in the lowest tertile. Though estimates were imprecise, there was little evidence that associations between maternal urinary phthalate concentrations and percent fat mass were modified by child’s sex.

**Conclusions:**

Prenatal phthalate exposures were not associated with increased body fat among children 4–9 years of age, though high prenatal DEHP exposure may be associated with lower fat mass in childhood.

**Citation:**

Buckley JP, Engel SM, Mendez MA, Richardson DB, Daniels JL, Calafat AM, Wolff MS, Herring AH. 2016. Prenatal phthalate exposures and childhood fat mass in a New York City cohort. Environ Health Perspect 124:507–513; http://dx.doi.org/10.1289/ehp.1509788

## Introduction

Childhood obesity prevalence has increased substantially in the past several decades (Ogden and Carroll 2010). In the United States, 16.9% of children 2–19 years of age are obese (Ogden et al. 2014). Compared with normal-weight children, obese children are at greater risk of becoming obese adults (Freedman et al. 2005), with associated risks of diabetes, heart disease, cancer, and many other ailments. Evidence suggests that factors in addition to energy balance have contributed to the obesity epidemic (Keith et al. 2006). The concomitant increase in obesity rates with increased production and use of synthetic chemicals in consumer products has led to a search for environmental causes of obesity (Baillie-Hamilton 2002). Xenobiotic chemicals, such as endocrine disruptors, are thought to be “environmental obesogens” that promote obesity through dysregulation of lipid metabolism and adipogenesis (Grün and Blumberg 2006).

Phthalates, synthetic chemicals with endocrine-disrupting properties, are hypothesized to be obesogens (Desvergne et al. 2009; Kim and Park 2014). Human exposure to phthalates is ubiquitous, arising from contact with a wide range of consumer products, including building materials, medical devices, pharmaceuticals, toys, food packaging, cosmetics, and fragrances (Schettler 2006). Phthalate metabolites have been detected in amniotic fluid (Silva et al. 2004; Wittassek et al. 2009), and early-life phthalate exposures have been associated with health outcomes in childhood, including physical and neurological development, allergic diseases, and anogenital distance in boys (reviewed by Braun et al. 2013). Dibutyl (DnBP), di-iso-butyl (DiBP), di-iso-nonyl (DiNP), benzylbutyl (BzBP), and di(2-ethylhexyl) (DEHP) phthalates exhibit anti-androgenic activity (National Research Council 2008), and phthalate associations for some developmental end points have been reported to vary by sex (Engel et al. 2009; Swan et al. 2010; Wolff et al. 2008).

Phthalate exposures during gestation are hypothesized to affect production and accumulation of body fat by modifying peroxisome proliferator–activated receptors (PPARs), associated with adipogenesis, lipid and carbohydrate metabolism, and androgen levels, or by disrupting thyroid hormone function (Desvergne et al. 2009; Kim and Park 2014). Potential effects of anti-androgenic phthalates may differ between girls and boys given that sex steroids are associated with sexually dimorphic development of adipose tissue (Grün and Blumberg 2009).

Although a number of studies, primarily cross-sectional, have reported associations between urinary phthalate metabolite concentrations and body size in children (recently reviewed by Goodman et al. 2014), only one previous study has examined gestational exposure, a hypothesized critical window for an effect of phthalates on obesity. Valvi et al. (2015) assessed associations between prenatal phthalate exposures and childhood body size in a Spanish birth cohort and reported sex-specific associations of high-, but not low-, molecular-weight phthalates with infant weight gain and body mass index (BMI) *z*-scores at ages 1, 4, and 7 years (inverse among boys, positive among girls). However, BMI is a measure of excess weight for height and does not distinguish between lean and fat mass (Freedman and Sherry 2009). To address this gap, we assessed the associations between third-trimester urinary phthalate metabolite concentrations and body composition estimates of percent fat mass at ages 4–9 years in a prospective birth cohort.

## Methods

*Study population.* The Mount Sinai Children’s Environmental Health Study enrolled 479 primiparous women with singleton pregnancies from the Mount Sinai Diagnostic and Treatment Center and two adjacent private practices in New York City between 1998 and 2002. Women delivered at the Mount Sinai Medical Center, and 75 women were subsequently excluded for reasons described elsewhere (Engel et al. 2007). The final birth cohort consists of 404 infants for whom birth data were available. Children were invited to return for three follow-up visits scheduled at approximately ages 4–5.5 (mean, 4.9), 6 (mean, 6.2), and 7–9 (mean, 7.8) years, hereafter referred to as visits 1, 2, and 3, respectively.

Of the 404 infants in the birth cohort, 382 had prenatal phthalate metabolite concentrations measured in maternal urine. Following previous work (Wolff et al. 2008), we excluded two observations obtained from a very dilute urine (< 10 mg/dL creatinine) because of the potential for inaccurate biomarker measurements. Of the remaining 380 infants, the present analysis includes 180 with at least one outcome measurement at follow-up (363 total visits for percent fat mass, 364 total visits for supplementary BMI analysis).

Women provided written informed consent before participation and children ≥ 7 years of age provided assent. The study received approval from the Mount Sinai School of Medicine Institutional Review Board. The involvement of the Centers for Disease Control and Prevention (CDC) laboratory was determined not to constitute engagement in human subjects research. The University of North Carolina at Chapel Hill Institutional Review Board approved the present analysis.

*Phthalate exposures.* Mothers provided a spot urine sample between 25 and 40 weeks’ gestation (mean, 31.5 weeks). Samples were analyzed at the CDC laboratory for the following phthalate metabolites: monoethyl phthalate [MEP, a metabolite of diethyl phthalate (DEP)], mono-*n*-butyl phthalate (MnBP, a metabolite of DnBP), monoisobutyl phthalate (MiBP, a metabolite of DiBP), mono(3-carboxypropyl) phthalate (MCPP, a nonspecific metabolite of several high molecular weight phthalates and a minor metabolite of DnBP), monobenzyl phthalate (MBzP, a metabolite of BzBP), and four metabolites of DEHP: mono(2-ethylhexyl) phthalate (MEHP), mono(2-ethyl-5-hydroxyhexyl) phthalate (MEHHP), mono(2-ethyl-5-oxohexyl) phthalate (MEOHP), and mono(2-ethyl-5-carboxypentyl) phthalate (MECPP). Laboratory and quality control methods have been reported previously (Kato et al. 2005). Correction factors were applied to MBzP (0.72) and MEP (0.66) concentrations and limits of detection (LOD) to adjust for inaccuracies in analytical standards (CDC 2012). Distributions of prenatal phthalate metabolite concentrations were similar among children with and without a follow-up visit (Engel et al. 2010).

*Outcome assessment.* At each follow-up visit, we measured children in bare or stocking feet wearing a pediatric gown or light clothing. We assessed weight and body composition via bioelectrical impedance analysis using a pediatric Tanita scale (model TBF-300; Tanita Corporation of America). We obtained duplicate measures of height using a stadiometer, and collected a third measure if the difference between the first two measures exceeded 2.0 cm.

Using the fat mass estimates reported by the Tanita scale, we calculated percent fat mass as (fat mass/weight) × 100. Because the Tanita equations for fat mass have not been validated for children < 7 years of age, we tested two alternative equations validated for estimating fat mass from bioelectrical impedance measures in children (Clasey et al. 2011; Horlick et al. 2002). The Horlick et al. (2002) equation performed poorly in this sample and yielded many percent fat mass values that exceeded total mass (data not shown). Percent fat mass values estimated with the Clasey et al. (2011) equation were highly correlated with the Tanita values (Pearson *R* ^2^ = 0.97) but yielded some implausible estimates. Thus, we used the Tanita proprietary equations. For supplementary analyses, we also calculated BMI as weight (kilograms)/height (meters)^2^ and determined age- and sex-standardized BMI *z*-scores using a CDC SAS macro (CDC 2004).

*Covariates.* We collected covariate data from mothers during a 2-hr structured interview at enrollment. We ascertained pregnancy and delivery characteristics from a computerized perinatal database at Mount Sinai Hospital. We calculated adequacy of gestational weight gain as the ratio of observed gestational weight gain (last pregnancy weight minus self-reported prepregnancy weight) to expected gestational weight gain based on the 2009 Institute of Medicine recommendations × 100 (IOM/NRC 2009). For tabular presentation, we categorized gains as less than recommended (< 86%), recommended (86–120%), or more than recommended (> 120%) (Bodnar et al. 2011). We classified physical activity at each follow-up visit as active if the parent/caretaker reported the child was “active most of the time” or inactive if the child was “active some of the time” or “hardly at all.”

*Statistical analysis.* We used a Bayesian modeling framework to assess associations between prenatal phthalate metabolite concentrations and percent fat mass. We selected this approach to estimate associations while simultaneously addressing several potential biases by *a*) imputing metabolite concentrations below the LOD, *b*) stabilizing estimates from multiple metabolite models, *c*) imputing missing covariate data, and *d*) examining potentially informative loss to follow-up.

First, we accounted for phthalate metabolite concentrations below the LOD by imputing values from a truncated normal distribution (Carmichael et al. 2010; Uh et al. 2008). At each iteration of the Markov chain Monte Carlo (MCMC) algorithm, we imputed natural log values of each metabolite from a truncated normal distribution with parameters defined as the mean and standard deviation (SD) of the observed distribution, a lower bound of 0, and an upper bound set equal to the LOD (WinBUGS package djl.dnorm.trunc). Because DEHP metabolites (MECPP, MEHHP, MEHP, MEOHP) represent exposure from the same sources and are highly correlated (Wolff et al. 2008), we examined them as a molar sum (ΣDEHP) that was computed using component metabolite values at each MCMC iteration. We standardized the natural log of each phthalate metabolite or sum to its mean and SD to facilitate comparison of estimated relative phthalate effect sizes in relation to their distribution in the study population.

Phthalate metabolite concentrations are correlated because they have common parent phthalates and source products, suggesting that estimates of association between percent fat mass and an individual phthalate metabolite may be confounded by other metabolites. Therefore, we estimated associations in multiple metabolite hierarchical models within our Bayesian framework. We gave the beta coefficient of each standardized phthalate metabolite an independent normal prior distribution with a mean of zero and variance of 1/τ^2^ (MacLehose et al. 2007). We selected a value of τ that reflects our prior belief that 95% of the effects of a SD difference in natural log phthalate metabolite concentration are within approximately ± 1 SD of the mean percent fat mass in the study population (τ = 1/16). As a sensitivity analysis, we estimated associations using single metabolite models that specified the same prior distributions for phthalate beta coefficients but included only one metabolite at a time.

We adjusted for potential confounding variables identified using directed acyclic graphs (see Supplemental Material, Figure S1). Demographic and socioeconomic characteristics included maternal race/ethnicity (non-Hispanic white/non-Hispanic black/Hispanic), age, education (less than college/college degree or more), and work status during pregnancy (employed/student or homemaker). We adjusted for maternal body size characteristics (prepregnancy BMI and adequacy of gestational weight gain), maternal smoking during pregnancy (yes/no), and breastfeeding (ever/never) to account for early-life factors that are associated with childhood overweight status (Weng et al. 2012). We adjusted for calendar date of urine collection to account for temporal trends in phthalate exposure and prevalence of childhood obesity. Additionally, we adjusted for natural log creatinine (to account for urine dilution), child’s sex, months of age at follow-up, and a product term between child’s sex and months of age at follow-up. We also included strong predictors of the outcome, including maternal height and child physical activity at follow-up (active/inactive), to improve precision. We evaluated child’s sex as an effect measure modifier by including a product term between sex and each metabolite. We standardized continuous covariates (maternal age, prepregnancy BMI, maternal height, adequacy of gestational weight gain, calendar date of urine collection, natural log creatinine, and age at follow-up) as (*x* – *μ_x_*)/(2σ*_x_*) to improve MCMC convergence and facilitate comparisons of estimated effect sizes with dichotomous covariates (Gelman et al. 2013). We used higher-order polynomials to model maternal age (cubic), maternal prepregnancy BMI (quadratic), adequacy of gestational weight gain (cubic), and natural log creatinine (quadratic) based on visual assessment of associations between covariates and percent fat mass using generalized additive models (SAS PROC GAM; SAS Institute Inc.) and likelihood ratio tests assessing improvement in model fit. We specified independent null-centered priors with τ = 1/64 for each covariate, reflecting our prior belief that 95% of the effects of binary covariates (or a 2-SD change in continuous covariates) are within ± 2 SDs of the mean percent fat mass in the study population.

Again taking advantage of the Bayesian framework for multiple imputation, we imputed missing values for adequacy of gestational weight gain (*n* = 22), breastfeeding (*n* = 1), and physical activity at follow-up (*n* = 3 children with eight visits) within the MCMC procedure under the assumption that values are missing at random. Because missing adequacy of gestational weight gain was attributable to missing last pregnancy weights, we modeled last pregnancy weight as a normally distributed random variable conditional on maternal education, race/ethnicity, child’s sex, smoking during pregnancy, work status during pregnancy, maternal age at delivery, birth weight, maternal height, gestational age, maternal first pregnancy weight, and maternal prepregnancy BMI. At each iteration of the MCMC algorithm, adequacy of gestational weight gain was calculated using the imputed last pregnancy weight. We modeled breastfeeding using a logistic model conditional on maternal education, race/ethnicity, child’s sex, smoking during pregnancy, work status during pregnancy, maternal age at delivery, adequacy of gestational weight gain, maternal prepregnancy BMI, and birth weight. We modeled physical activity at each follow-up visit using a logistic mixed effects model with random intercepts, conditional on race/ethnicity, maternal prepregnancy BMI, smoking during pregnancy, age at follow-up (months), birth weight, and child’s sex. We gave beta coefficients in the imputation models independent, null-centered priors with τ = 1, reflecting our prior belief that 95% of the effects of binary covariates (or a 2-SD change in continuous covariates) are within ± 2 SDs of the mean last pregnancy weight or within an odds ratio of 0.14–7.1 for breastfeeding and physical activity.

We assessed associations of third-trimester maternal urinary phthalate metabolite concentrations with percent fat mass using linear mixed-effects regression models with random intercepts to account for multiple observations per child. We estimated posterior mean beta coefficients and 95% credible intervals (CI) per SD increase in natural log phthalate metabolite concentrations. The interpretation of a CI is more intuitive than that of a confidence interval from a conventional analysis; specifically, given the data and the model, there is a 95% chance that the true value is within this interval. In models examining modification by child’s sex, we included product terms between sex and each metabolite, as well as between sex and natural log creatinine, and considered there to be meaningful modification if the 80% CI did not cross the null value. To assess potential nonlinear dose–response relationships, we also fit models with indicator variables for the second and third tertile categories of each metabolite (tertile classification was based on metabolite distributions among the 180 children in the sample). To account for urine dilution in these analyses, we categorized exposure using creatinine-corrected concentrations (micrograms per gram creatinine for metabolites or micromoles per gram creatinine for ΣDEHP) and did not include natural log creatinine as a covariate.

To assess a second measure of childhood adiposity and facilitate comparisons to studies without body composition data, we also estimated associations between phthalate metabolite concentrations and BMI *z*-scores using the statistical approach described above.

We ran each of the final Bayesian models for 50,000 iterations after an initial 10,000 iteration burn-in, and assessed model convergence using standard diagnostic measures described elsewhere (Gelman et al. 2013). We created causal diagrams using DAGitty version 2.2 (Textor et al. 2011), conducted descriptive analyses in SAS version 9.3 (SAS Institute Inc.), and performed Bayesian modeling in WinBUGS version 1.4.3 (MRC Biostatistics Unit).

*Sensitivity analysis for loss to follow-up.* Our primary analyses of children with at least one follow-up visit (*n* = 180) assumed that responses were missing-at-random conditional on observed outcomes and covariates. Because there were some differences in the distribution of observed variables between the birth cohort and the follow-up sample (Engel et al. 2010) and because the probability of a missing fat mass measure may depend upon its true (unobserved) value, we assessed the sensitivity of our findings to potential selection bias. We used a selection model approach (Little and Rubin 2002) to model the association between phthalate exposures and percent fat mass among all 380 children with measured prenatal phthalate metabolite concentrations, under a potentially nonignorable (missing not at random) missing data mechanism. For this analysis, we jointly fit our model for percent fat mass with a logistic model for a binary indicator of whether the outcome value was missing at each follow-up visit. The missing data indicator model was dependent on the possibly unobserved percent fat mass value as well as covariates either observed or expected to be associated with loss to follow-up (maternal age at delivery, race/ethnicity, prepregnancy BMI, adequacy of gestational weight gain, child’s sex, months of age at follow-up). Because results from selection models are sensitive to the assumed missing data mechanism (Little 1995), we explored models with additional covariates in the missing data indicator model (maternal smoking during pregnancy, breastfeeding, maternal education, work status during pregnancy, birth weight) and varied parameterization of continuous variables. Beta coefficients were given null-centered, independent priors with τ = 2 to reflect our prior belief that 95% of the effects of a binary covariate (or a 2-SD change in continuous covariates) are within an odds ratio of 0.25–4. We ran 10 chains for inference from the MCMC procedure and compared results to models that assumed dropout was missing at random.

## Results

Although baseline characteristics of children included in the study sample were similar to those in the birth cohort, there were minor differences (Table 1). For example, children of mothers with younger maternal age, underweight prepregnancy BMI, and less than recommended gestational weight gain were more likely to be lost to follow-up. All phthalate metabolites were detected in > 90% of third-trimester maternal urine samples and geometric mean concentrations were somewhat higher than reported among females ≥ 6 years of age in the 2001–2002 National Health and Nutrition Examination Survey (CDC 2015), a nationally representative sample of U.S. females during a similar time frame (Table 2).

**Table 1:** Characteristics of participants at birth and follow-up, Mount Sinai Children’s Environmental Health Study 1998–2002.

Characteristic	Birth cohort *n* (%)	Study sample *n* (%)
Total (*n*)	404	180
Race/ethnicity
Non-Hispanic white	86 (21.3)	34 (18.9)
Non-Hispanic black	112 (27.7)	51 (28.3)
Hispanic or other	206 (51)	95 (52.8)
Maternal age at delivery (years)
< 20	142 (35.2)	56 (31.1)
20–24	132 (32.7)	60 (33.3)
25–29	44 (10.9)	26 (14.4)
≥ 30	86 (21.3)	38 (21.1)
Maternal education (≥ college degree)	100 (24.8)	40 (22.2)
Maternal work status (employed)	235 (58.2)	107 (59.4)
Maternal smoking during pregnancy	67 (16.6)	31 (17.2)
Maternal prepregnancy BMI (kg/m^2^)
< 18.5	82 (20.3)	10 (5.6)
18.5–24.9	215 (53.2)	111 (61.7)
25–29.9	72 (17.8)	42 (23.3)
≥ 30	35 (8.7)	17 (9.4)
Maternal height (m) (mean ± SD)	1.63 ± 0.07	1.62 ± 0.08
Adequacy of gestational weight gain
Less than recommended	44 (12.3)	14 (8.9)
Recommended	75 (21)	40 (25.3)
More than recommended	238 (66.7)	104 (65.8)
Missing	47	22
Year of maternal urine collection
1998	84 (21.9)	34 (18.9)
1999	127 (33.2)	55 (30.6)
2000	134 (35)	72 (40)
2001	35 (9.1)	18 (10)
2002	3 (0.8)	1 (0.6)
Urine not collected	21	0
Child’s sex (male)	222 (55)	98 (54.4)
Breastfed
Ever	206 (63)	113 (63.1)
Never	121 (37)	66 (36.9)
Missing	77	1
Physical activity at follow-up^*a*^
Inactive		100 (56.5)
Active most of the time		77 (43.5)
Missing		3
^***a***^Proportion classified as inactive at any follow-up visit.		

**Table 2:** Distributions of phthalate metabolite concentrations (μg/L) in third trimester maternal urine samples (*n *= 180), Mount Sinai Children’s Environmental Health Study 1998–2002.

Metabolite (μg/L)	LOD	Percent detected	Geometric mean^*a*^	Minimum	25th percentile	75th percentile	Maximum	NHANES^*b*^
MEP	0.26	99.4	223	< LOD	87.1	525	29,528	115
MnBP	0.4	100	32.9	0.800	14.3	79.5	4,043	20.2
MiBP	0.26	97.8	5.83	< LOD	2.90	15.1	76.6	2.68
MCPP	0.16	97.8	2.87	< LOD	1.50	6.30	129	2.62
MBzP	0.08	99.4	14.1	< LOD	5.70	32.8	481	10.5
∑DEHP^*c*^	NA	NA	0.284	< LOD	0.125	0.530	19.9
MECPP	0.25	99.4	36.0	< LOD	15.1	72.7	2,055	31.9^*d*^
MEHHP	0.32	99.4	21.0	< LOD	8.80	41.3	2,051	18.3
MEHP	0.9	91.7	6.15	< LOD	3.00	14.2	478	4.23
MEOHP	0.45	99.4	18.7	< LOD	8.20	38.3	1,335	12.5
Abbreviations: NA, not available; NHANES, National Health and Nutrition Examination Survey. ^***a***^To compute the geometric mean, phthalate metabolite concentrations < LOD were replaced by LOD divided by the square root of 2. ^***b***^Geometric mean phthalate metabolite concentrations among 1,411 female NHANES 2001–2002 participants age ≥ 6 years (CDC 2015). ^***c***^∑DEHP is expressed as micromoles/L. ^***d***^MECPP was not included in NHANES 2001–2002; data are from NHANES 2003–2004 (*n *= 1,355).								

Twenty-one percent of the children were classified as obese during at least one follow-up visit (19% at visit 1, 14% at visit 2, and 22% at visit 3). Percent fat mass increased with age and differed by child’s sex, with a larger SD among girls than among boys at all ages (Table 3). As expected, BMI *z*-score distributions were less variable as they are age- and sex-standardized (see Supplemental Material, Table S1). Correlations between percent fat mass and BMI *z*-scores increased with age; Spearman *R* ^2^s were 0.75, 0.82, and 0.91 and the first, second, and third visits, respectively.

**Table 3:** Percent fat mass distributions in the Mount Sinai Children’s Environmental Health Study.*^a^*

Visit	Age (years) (mean ± SD	Overall		Girls		Boys	
		*n*	Mean ± SD	*n*	Mean ± SD	*n*	Mean ± SD
All visits	6.5 ± 1.3	363	18.4 ± 8.4	173	17.5 ± 9.8	190	19.3 ± 6.8
Visit 1	4.9 ± 0.4	97	15.3 ± 7.5	47	12.6 ± 8.8	50	17.8 ± 4.8
Visit 2	6.1 ± 0.2	117	17.5 ± 7.2	57	16.4 ± 8.6	60	18.5 ± 5.4
Visit 3	7.8 ± 0.8	149	21.2 ± 9.0	69	21.6 ± 9.8	80	20.9 ± 8.3
^***a***^Percent fat mass estimated using bioelectrical impedance analysis (Tanita TBF-300).							

**Figure 1:**
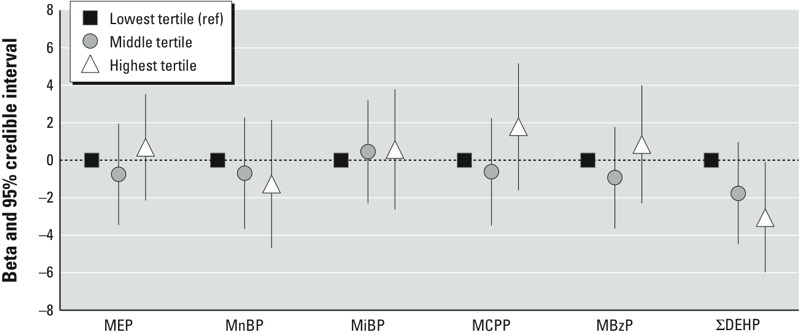
Adjusted associations between tertiles of third trimester maternal urinary phthalate metabolite concentrations and percent fat mass among children 4–9 years of age in the Mount Sinai Children’s Environmental Health Study. ref, reference.

Estimates of association between standardized natural log phthalate metabolite concentrations and percent fat mass are reported in Table 4. All 95% CIs included the null value and no associations were modified by child’s sex (i.e., all 80% CIs for phthalate × sex product terms included the null value), though estimates were imprecise. In models assessing metabolite tertiles, there was an inverse relationship for the association of ΣDEHP with percent fat mass (Figure 1). Compared with the lowest ΣDEHP tertile, fat mass was 1.77% (95% CI: –4.48, 0.97%) lower in the middle tertile and 3.06% (95% CI: –5.99, –0.09%) lower in the highest tertile. We found no evidence of associations between tertile categories of other metabolites and percent fat mass, nor evidence of differences in associations of phthalate tertiles with percent fat mass by child’s sex, though CIs were wide (see Supplemental Material, Table S2).

**Table 4:** Adjusted associations between third trimester maternal urinary phthalate metabolite concentrations and percent fat mass among children age 4–9 years in the Mount Sinai Children’s Environmental Health Study.

Metabolite	Overall	Girls	Boys^*a*^
MEP	0.12 (–1.34, 1.58)	–0.35 (–2.43, 1.75)	0.75 (–1.31, 2.80)
MnBP	–0.86 (–3.07, 1.36)	–0.34 (–3.71, 3.05)	–0.86 (–3.46, 1.74)
MiBP	0.34 (–1.54, 2.20)	1.04 (–1.36, 3.44)	–0.88 (–3.44, 1.68)
MCPP	0.63 (–1.55, 2.82)	1.21 (–1.44, 3.87)	–0.08 (–3.22, 3.03)
MBzP	0.67 (–1.31, 2.65)	0.62 (–1.77, 3.02)	0.98 (–2.17, 4.14)
∑DEHP	–0.89 (–2.24, 0.47)	–0.80 (–2.81, 1.23)	–0.64 (–2.46, 1.16)
Beta coefficients (95% credible intervals) per standard deviation increase in natural log phthalate metabolite concentrations were estimated in a multiple metabolite linear mixed-effects regression model. Sex-specific estimates were modeled by including product terms for phthalate × child’s sex. Estimates are adjusted for urine dilution and collection date; maternal race/ethnicity, age, education, work status, and smoking during pregnancy; maternal height and prepregnancy BMI; adequacy of gestational weight gain; breastfeeding; months of age and physical activity at follow-up; and, for overall models, child’s sex. ^***a***^Sex-specific estimates did not meet criteria for heterogeneity (i.e., 80% credible intervals for all phthalate × sex product terms included the null value).			

Results of sensitivity analyses are reported in Table 5. Associations estimated in multiple and single metabolite models were similar, though the multiple metabolite model beta coefficients tended to be slightly farther from the null and CIs were less precise. Results of the sensitivity analysis for loss to follow-up indicated that missing fat mass data may indeed be nonignorable (i.e., the probability of follow-up depends on the child’s potentially unobserved percent fat mass value). Children with more fat mass were less likely to be lost to follow-up: For each 1% increase in fat mass, the odds ratio for loss to follow-up was 0.81 (95% CI: 0.69, 0.94). However, associations between phthalate metabolites and percent fat mass estimated in a selection model accounting for nonignorable missing outcomes were similar to the primary analysis (Table 5). Varying parameters in the missing data indicator model did not alter results (data not shown).

**Table 5:** Sensitivity analyses for associations between third trimester maternal urinary phthalate metabolite concentrations and percent fat mass among children 4–9 years of age in the Mount Sinai Children’s Environmental Health Study.

Metabolite	Primary analysis^*a*^	Single metabolite models^*b*^	Loss to follow-up^*c*^
MEP	0.12 (–1.34, 1.58)	0.08 (–1.14, 1.30)	–0.12 (–1.62, 1.36)
MnBP	–0.86 (–3.07, 1.36)	–0.07 (–1.41, 1.27)	–0.93 (–3.15, 1.30)
MiBP	0.34 (–1.54, 2.20)	0.23 (–1.12, 1.52)	1.03 (–0.91, 2.97)
MCPP	0.63 (–1.55, 2.82)	0.30 (–1.05, 1.66)	0.40 (–1.81, 2.60)
MBzP	0.67 (–1.31, 2.65)	0.40 (–0.93, 1.74)	0.48 (–1.47, 2.47)
∑DEHP	–0.89 (–2.24, 0.47)	–0.57 (–1.79, 0.57)	–0.62 (–2.01, 0.81)
Beta coefficients (95% credible intervals) per SD increase in natural log phthalate metabolite concentrations, adjusted for urine dilution and collection date; maternal race/ethnicity, age, education, work status, and smoking during pregnancy; maternal height and prepregnancy BMI; adequacy of gestational weight gain; breastfeeding; months of age and physical activity at follow-up; and child’s sex. ^***a***^Associations among children with at least one follow-up visit (*n *= 180) estimated in a multiple metabolite linear mixed-effects regression model. ^***b***^Associations among children with at least one follow-up visit (*n *= 180) estimated in a separate linear mixed-effects regression model for each metabolite. ^***c***^Associations among all children in the birth cohort with measured phthalate metabolite concentrations (*n *= 380) estimated in a multiple metabolite linear mixed-effects regression model using a selection model for potentially nonignorable missing outcome data.			

Patterns of association between phthalate metabolite concentrations and BMI *z*-scores were generally consistent with our analyses of percent fat mass, though estimates were attenuated toward the null (see Supplemental Material, Table S3). Notably, we did not observe associations between ΣDEHP metabolite concentrations and BMI *z*-scores in continuous or tertile models (see Supplemental Material, Table S3).

## Discussion

In this prospective study of gestational phthalate exposures and adiposity in childhood, we did not observe associations between continuously modeled third trimester maternal urinary phthalate metabolite concentrations and percent fat mass. On average, children in the highest tertile of ΣDEHP metabolites had approximately 3% lower fat mass at ages 4–9 years than did children in the lowest tertile of ΣDEHP metabolites. We did not observe notable modification of associations by child’s sex, though there is limited ability to detect heterogeneity in this small sample. Findings were robust to adjustment for a comprehensive set of confounders and to a sensitivity analysis assessing bias from loss to follow-up.

Our findings suggest an anti-adipogenic effect of DEHP exposure, which is consistent with toxicological studies reporting that relatively high-dose postnatal DEHP exposure (doses equivalent to 2% of the animal’s body weight) induces PPARα-mediated reductions in body weight and fat mass in both rats (Itsuki-Yoneda et al. 2007) and mice (Xie et al. 2002). However, toxicological studies examining early-life exposure to DEHP or its hydrolytic metabolite, MEHP, have reported species- and dose-specific effects on subsequent body fat of offspring. Two studies of perinatal MEHP or DEHP exposure in mice reported increased body weight and fat deposition in offspring, though one study observed effects only at the lowest dose administered (0.05 but not 0.25 or 0.5 mg MEHP/kg/day) (Hao et al. 2012), and the other reported effects at both low and high doses (0.05 and 5 mg DEHP/kg/day) (Schmidt et al. 2012). Two studies of pregnant rats exposed to doses ranging from 1 to 400 mg DEHP/kg/day reported no difference in offspring total body weight (Campioli et al. 2014; Kobayashi et al. 2006). Interestingly, Feige et al. (2010) reported that although high dose DEHP exposure (500 mg/kg body mass/day) protected against diet-induced obesity via PPARα activation in wild-type mice, mice with humanized PPARα gained more weight and adipose tissue than untreated controls. Although the causes of species differences remain unclear, effects of DEHP on other, less understood pathways (e.g., liver metabolism, thyroid function, androgen activity) may play a role.

A study that examined DEHP metabolite concentrations in cord plasma in relation to repeated BMI measurements in the first year of life reported associations of one metabolite, MEOHP, with lower BMI in boys (de Cock et al. 2014). However, this study was based on a small sample at each time point (*n* < 10). Further, the use of phthalate metabolite concentrations in blood or blood products as exposure biomarkers is questionable (Calafat et al. 2013). Although the authors measured only secondary or oxidative metabolites, which may reflect true biological exposures, it is possible that cord plasma DEHP metabolites arise from hospital-based exposures to DEHP at the time of delivery (Yan et al. 2009). Valvi et al. (2015) examined associations between the average of first- and third-trimester maternal urinary phthalate metabolites concentrations and difference in weight gain *z*-score in the first 6 months, BMI *z*-scores at ages 1, 4, and 7 years, and waist-to-height ratio at ages 4 and 7 years in a Spanish birth cohort (*n* = 391). Associations between summed high-molecular-weight phthalates concentrations (four DEHP metabolites and MBzP) and infant weight gain and BMI *z*-scores were inverse among boys but positive among girls (supplementary analyses indicated these associations were attributable to ΣDEHP and not MBzP). Similar to our findings, Valvi et al. (2015) did not identify associations between prenatal concentrations of low-molecular-weight phthalate metabolites and childhood body size. Although we had low power to detect sex differences in associations of ΣDEHP metabolites with percent fat mass, estimates comparing the highest with lowest tertiles of ΣDEHP metabolites were very similar among boys (β: –2.99%; 95% CI: –7.10, 1.15%) and girls (β: –3.07%; 95% CI: –6.54, 0.41%).

A number of cross-sectional analyses have examined phthalate exposures and obesity, BMI, waist circumference, or related measures in humans (recently reviewed by Goodman et al. 2014). Many of these studies reported associations between phthalate metabolites and adiposity-related outcomes, with results often differing between groups defined by age, sex, race/ethnicity, or other characteristics. Such cross-sectional analyses are problematic because it is unclear whether phthalate exposures are causally related to obesity or phthalate body burden differs in obese individuals due to differences in exposure to phthalate sources, such as diet (Serrano et al. 2014), personal care products (Buckley et al. 2012), or medications (Hernández-Díaz et al. 2013). In addition, these analyses did not assess exposures during gestation, a hypothesized critical window for an effect of phthalates on pathways related to development of obesity.

Phthalates have relatively short half-lives and are excreted in urine in < 24 hr (Koch et al. 2005), and exposures are likely episodic in nature, so that a single third-trimester urine sample may not be representative of exposure during all of gestation. However, fetal growth and adipocyte replication is rapid during the third trimester, indicating that it is a relevant exposure period for fat development (Dietz 1994). Additionally, studies of repeat urinary phthalate measures during pregnancy suggest that concentrations of phthalate metabolites in a single spot urine sample may be moderately predictive of DEP, DBP, DiBP, and BzBP concentrations throughout pregnancy, with greater variability over time for DEHP (Adibi et al. 2008; Braun et al. 2012; Ferguson et al. 2014).

Food packaging may account for more than half of total DEHP exposure (Rudel et al. 2011), and DEHP has consistently been detected in foods such as dairy, poultry, and cooking oils (Serrano et al. 2014). Although we adjusted for maternal prepregnancy BMI and adequacy of gestational weight gain, there may be residual confounding by maternal food preferences that are both a source of prenatal phthalate exposures and a cause of increased fat mass in offspring. To explain the inverse association we observed between ΣDEHP and percent fat mass, foods that are part of a healthy family diet would also have to contain more DEHP. We also did not adjust for childhood calorie intake, which is a strong predictor of body fat but is not a confounder in this study because it occurs temporally after maternal third-trimester phthalate exposure. Furthermore, if phthalate exposures increase adiposity through an effect on the child’s ability to regulate hunger, caloric intake may be a mediator of associations between prenatal phthalate exposures and childhood fat mass.

Although body composition equations built into the Tanita scale are not validated in children < 7 years, percent fat mass values estimated using an equation validated for this age group were strongly correlated with the values estimated by the proprietary equation. Furthermore, associations between phthalate metabolite concentrations and percent fat mass were consistent across study visits (data not shown), and patterns of association were similar to those for BMI *z*-score associations. Our sample size was not adequate to examine potential critical windows of susceptibility. Future studies may consider exploring relationships between phthalate exposures and obesity with respect to differing growth trajectories, timing of adiposity rebound, or pubertal onset. In addition, we did not have information on postnatal phthalate exposures and could not examine whether childhood phthalate exposures are also related to subsequent body fat. Finally, the role of other environmental obesogens as potential confounders or effect modifiers of associations between phthalates and adiposity should be explored in future analyses.

This analysis has several important strengths. First, we examined exposure to phthalates during the hypothesized critical window for developmental programming of obesity. Second, we incorporated repeated measures of fat mass in children. Third, we adjusted for many important confounders including maternal anthropometric characteristics and multiple measures of socioeconomic status. Fourth, we used a Bayesian framework to incorporate prior information and account for potential confounding by correlated metabolite concentrations. Finally, we investigated and accounted for missing data in exposures (values < LOD), covariates, and outcomes.

## Conclusions

In this prospective study, prenatal exposures to phthalates were not associated with increased body fat in children age 4–9 years. In tertile models, children in the highest tertile of maternal third-trimester urinary concentrations of ΣDEHP metabolites had lower percent fat mass at follow-up compared to those in the lowest tertile. Although a substantial cross-sectional literature has assessed phthalate metabolite concentrations and obesity-related outcomes, larger prospective studies examining prenatal exposures are needed to replicate these findings.

## Supplemental Material

(595 KB) PDF
