# Supplementary material for: Prenatal Phthalate Exposures and Childhood Fat Mass in a New York City Cohort
Source: Environ Health Perspect. 2015 Aug 25;124(4):507–13. doi: 10.1289/ehp.1509788 (PMC4829985; doi:10.1289/ehp.1509788)
Supplement: (595 KB) PDF [file ehp.1509788.s001.acco.pdf]

Environ Health Perspect
DOI: 10.1289/ehp.1509788

Note to Readers: *EHP* strives to ensure that all journal content is accessible to all readers. However, some figures and Supplemental Material published in *EHP* articles may not conform to 508 standards due to the complexity of the information being presented. If you need assistance accessing journal content, please contact ehp508@niehs.nih.gov. Our staff will work with you to assess and meet your accessibility needs within 3 working days.

Supplemental Material

Prenatal Phthalate Exposures and Childhood Fat Mass in a New York City Cohort

Jessie P. Buckley, Stephanie M. Engel, Michelle A. Mendez, David B. Richardson, Julie L. Daniels, Antonia M. Calafat, Mary S. Wolff, and Amy H. Herring

Table of Contents

Figure S1. Directed acyclic graph of associations between prenatal phthalate exposures, covariates, and child fat mass in the Mount Sinai Children's Environmental Health Study

Table S1. Age- and sex- standardized body mass index z-score distributions in the Mount Sinai Children's Environmental Health Study

Table S2. Adjusted associations between third trimester maternal urinary phthalate metabolite concentrations and percent fat mass among children aged 4 to 9 years in the Mount Sinai Children's Environmental Health Study

Table S3. Adjusted associations between third trimester maternal urinary phthalate metabolite concentrations and age- and sex- standardized body mass index z-scores among children aged 4 to 9 years in the Mount Sinai Children's Environmental Health Study

Figure S1. Directed acyclic graph of associations between prenatal phthalate exposures, covariates, and child fat mass in the Mount Sinai Children's Environmental Health Study

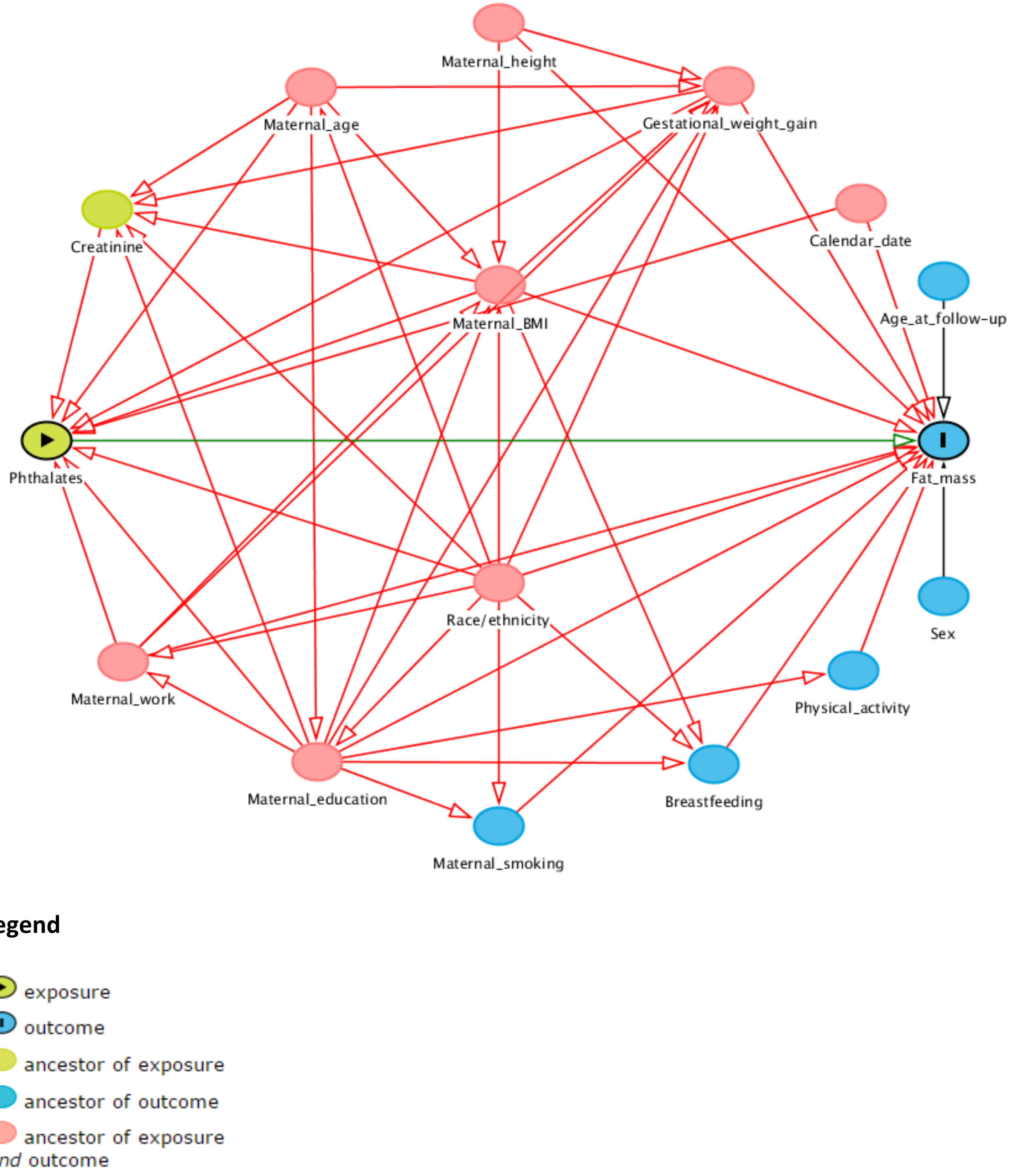

Table S1. Age- and sex- standardized body mass index z-score distributions in the Mount Sinai Children's Environmental Health Study

Visit	Mean age (years) ± SD	Overall		Girls		Boys	
		n	Mean ± SD	n	Mean ± SD	n	Mean ± SD
All visits	6.5 ± 1.3	364	0.56 ± 1.15	174	0.50 ± 1.10	190	0.62 ± 1.20
Visit 1	4.9 ± 0.4	99	0.51 ± 1.18	49	0.51 ± 1.07	50	0.52 ± 1.29
Visit 2	6.1 ± 0.2	117	0.50 ± 1.04	57	0.41 ± 1.04	60	0.59 ± 1.05
Visit 3	7.8 ± 0.8	148	0.63 ± 1.21	68	0.57 ± 1.17	80	0.69 ± 1.25

Standard deviation (SD)

Environ Health Perspect
DOI: 10.1289/ehp.1509788

Table S2. Adjusted associations between third trimester maternal urinary phthalate metabolite concentrations and percent fat mass among children aged 4 to 9 years in the Mount Sinai Children's Environmental Health Study

Metabolite	Exposure metric	Exposure unit[a]	Overall		Girls		Boys[d]	
			n[b]	β (95% CI)[c]	*n*[b]	β (95% CI)[c]	*n*[b]	β (95% CI)[c]
MEP	Lowest tertile	<186	126	ref	47	ref	75	ref
	Middle tertile	186–546	116	-0.75 (-3.45, 1.95)	56	-1.66 (-4.95, 1.69)	64	-0.20 (-3.81, 3.43)
	Highest tertile	>546	121	0.69 (-2.15, 3.53)	70	-0.38 (-3.77, 3.04)	51	2.46 (-1.49, 6.36)
	Continuous	SD	363	0.12 (-1.34, 1.58)	173	-0.35 (-2.43, 1.75)	190	0.75 (-1.31, 2.80)
MnBP	Lowest tertile	<32.0	132	ref	49	ref	77	ref
	Middle tertile	32.0–64.6	106	-0.69 (-3.66, 2.27)	55	1.00 (-2.64, 4.60)	61	-1.80 (-5.68, 2.10)
	Highest tertile	>64.6	125	-1.27 (-4.69, 2.16)	69	-0.43 (-4.37, 3.49)	52	-1.71 (-6.19, 2.82)
	Continuous	SD	363	-0.86 (-3.07, 1.36)	173	-0.34 (-3.71, 3.05)	190	-0.86 (-3.46, 1.74)
MiBP	Lowest tertile	<6.0	122	ref	39	ref	93	ref
	Middle tertile	6.0–12.2	120	0.46 (-2.30, 3.20)	61	0.55 (-2.94, 4.02)	45	0.71 (-2.93, 4.32)
	Highest tertile	>12.2	121	0.57 (-2.63, 3.79)	73	1.01 (-2.67, 4.69)	52	-0.07 (-4.44, 4.32)
	Continuous	SD	363	0.34 (-1.54, 2.20)	173	1.04 (-1.36, 3.44)	190	-0.88 (-3.44, 1.68)
MCPP	Lowest tertile	<2.8	131	ref	49	ref	79	ref
	Middle tertile	2.8-6.2	111	-0.61 (-3.48, 2.24)	74	-0.50 (-3.87, 2.90)	52	-0.72 (-4.60, 3.18)
	Highest tertile	>6.2	121	1.79 (-1.61, 5.17)	50	2.06 (-1.78, 5.88)	59	1.64 (-2.98, 6.32)
	Continuous	SD	363	0.63 (-1.55, 2.82)	173	1.21 (-1.44, 3.87)	190	-0.08 (-3.22, 3.03)
MBzP	Lowest tertile	<12.7	128	ref	52	ref	79	ref
	Middle tertile	12.7–30.5	126	-0.93 (-3.65, 1.77)	57	-2.19 (-5.70, 1.34)	54	-0.72 (-4.35, 2.92)
	Highest tertile	>30.5	109	0.84 (-2.29, 4.01)	64	1.63 (-1.89, 5.20)	57	-0.79 (-5.28, 3.74)
	Continuous	SD	363	0.67 (-1.31, 2.65)	173	0.62 (-1.77, 3.02)	190	0.98 (-2.17, 4.14)
∑DEHP	Lowest tertile	<0.23	120	ref	44	ref	76	ref
	Middle tertile	0.23–0.60	120	-1.77 (-4.48, 0.97)	61	-1.53 (-4.96, 1.88)	59	-1.79 (-5.34, 1.75)
	Highest tertile	>0.60	123	-3.06 (-5.99, -0.09)	68	-3.07 (-6.54, 0.41)	55	-2.99 (-7.10, 1.15)
	Continuous	SD	363	-0.89 (-2.24, 0.47)	173	-0.80 (-2.81, 1.23)	190	-0.64 (-2.46, 1.16)

Referent category (ref), standard deviation (SD)

[a] Tertile cut points are expressed as micrograms per gram creatinine (or micromoles per gram creatinine for ∑DEHP).

[b] Number of follow-up visits.

[c] Beta coefficients (95% credible intervals) were estimated in multiple metabolite linear mixed effects regression models adjusted for urine collection date; maternal race/ethnicity, age, education, work status, and smoking during pregnancy; maternal height and pre-pregnancy body mass index; adequacy of gestational weight gain; breastfeeding; months of age and physical activity at follow-up; and, for overall models, child's sex. Models of continuous metabolite concentrations were adjusted for natural log creatinine. Sex-specific estimates were estimated in models including a product term between child's sex and each metabolite or ∑DEHP.

[d] Sex-specific estimates did not meet criteria for heterogeneity (i.e., 80% credible intervals for all phthalate*sex product terms included the null value).

Table S3. Adjusted associations between third trimester maternal urinary phthalate metabolite concentrations and age- and sex-standardized body mass index z-scores among children aged 4 to 9 years in the Mount Sinai Children's Environmental Health Study

Metabolite	Exposure metric	Exposure unit[a]	Overall		Girls		Boys	
			n[b]	β (95% CI)[c]	*n*[b]	β (95% CI)[c]	*n*[b]	β (95% CI)[c]
MEP	Lowest tertile	<186	126	ref	47	ref	75	ref
	Middle tertile	186–546	116	0.16 (-0.23, 0.55)	57	-0.02 (-0.48, 0.44)	64	0.34 (-0.18, 0.87)
	Highest tertile	>546	122	0.17 (-0.24, 0.58)	70	0.13 (-0.35, 0.61)	51	0.23 (-0.33, 0.79)
	Continuous	SD	364	0.05 (-0.16, 0.27)	174	0.02 (-0.28, 0.33)	190	0.15 (-0.15, 0.45)
MnBP	Lowest tertile	<32.0	131	ref	49	ref	77	ref
	Middle tertile	32.0–64.6	108	-0.17 (-0.59, 0.25)	55	0.07 (-0.43, 0.57)[d]	61	-0.41 (-0.96, 0.15)[d]
	Highest tertile	>64.6	125	-0.19 (-0.67, 0.30)	70	-0.16 (-0.69, 0.38)	52	-0.25 (-0.90, 0.39)
	Continuous	SD	364	-0.13 (-0.45, 0.19)	174	-0.10 (-0.57, 0.37)	190	-0.15 (-0.53, 0.23)
MiBP	Lowest tertile	<6.0	122	ref	38	ref	93	ref
	Middle tertile	6.0–12.2	121	0.10 (-0.30, 0.50)	63	0.16 (-0.33, 0.65)	45	0.05 (-0.47, 0.57)
	Highest tertile	>12.2	121	0.03 (-0.42, 0.49)	73	0.06 (-0.44, 0.57)	52	-0.05 (-0.68, 0.58)
	Continuous	SD	364	-0.02 (-0.29, 0.25)	174	0.04 (-0.30, 0.38)	190	-0.13 (-0.51, 0.24)
MCPP	Lowest tertile	<2.8	131	ref	49	ref	79	ref
	Middle tertile	2.8-6.2	112	0.11 (-0.31, 0.52)	74	0.11 (-0.37, 0.59)	52	0.19 (-0.37, 0.74)
	Highest tertile	>6.2	121	0.32 (-0.16, 0.79)	51	0.35 (-0.18, 0.87)	59	0.40 (-0.26, 1.05)
	Continuous	SD	364	0.14 (-0.17, 0.46)	174	0.16 (-0.22, 0.54)	190	0.16 (-0.29, 0.61)
MBzP	Lowest tertile	<12.7	128	ref	52	ref	79	ref
	Middle tertile	12.7–30.5	126	-0.18 (-0.56, 0.22)	58	-0.23 (-0.72, 0.26)	54	-0.23 (-0.75, 0.29)
	Highest tertile	>30.5	110	-0.20 (-0.65, 0.25)	64	-0.06 (-0.56, 0.44)	57	-0.38 (-1.02, 0.26)
	Continuous	SD	364	-0.02 (-0.32, 0.27)	174	0.03 (-0.33, 0.38)	190	-0.11 (-0.56, 0.34)
∑DEHP	Lowest tertile	<0.23	120	ref	44	ref	76	ref
	Middle tertile	0.23–0.60	120	-0.10 (-0.49, 0.29)	61	-0.17 (-0.65, 0.30)	59	0.01 (-0.50, 0.52)
	Highest tertile	>0.60	124	-0.13 (-0.55, 0.29)	69	-0.13 (-0.60, 0.35)	55	-0.08 (-0.66, 0.51)
	Continuous	SD	364	-0.05 (-0.25, 0.15)	174	-0.05 (-0.34, 0.24)	190	-0.01 (-0.28, 0.26)

Referent category (ref), standard deviation (SD)

[a] Tertile cut points are expressed as micrograms per gram creatinine (or micromoles per gram creatinine for ∑DEHP).

[b] Number of follow-up visits.

[c] Beta coefficients (95% credible intervals) were estimated in multiple metabolite linear mixed effects regression models adjusted for urine collection date; maternal race/ethnicity, age, education, work status, and smoking during pregnancy; maternal height and pre-pregnancy body mass index; adequacy of gestational weight gain; breastfeeding; months of age and physical activity at follow-up; and, for overall models, child's sex. Models of continuous metabolite concentrations were adjusted for natural log creatinine. Sex-specific estimates were estimated in models including a product term between child's sex and each metabolite or ∑DEHP.

[d] Sex-specific estimates met criteria for heterogeneity (i.e., 80% credible interval for phthalate*sex product term excluded the null value).
